# Shared plasma metabolomic profiles of cognitive and mobility decline predict future dementia

**DOI:** 10.1007/s11357-024-01228-7

**Published:** 2024-06-03

**Authors:** Qu Tian, Shanshan Yao, Megan M. Marron, Erin E. Greig, Supriya Shore, Luigi Ferrucci, Ravi Shah, Venkatesh L. Murthy, Anne B. Newman

**Affiliations:** 1 Longitudinal Studies Section, National Institute on Aging, 251 Bayview Blvd M04B332, Baltimore, MD 21224 USA; 2 Department of Epidemiology, University of Pittsburgh, Pittsburgh, PA USA; 3 University of Michigan, Ann Arbor, MI USA; 4 Vanderbilt University Medical Center, Nashville, TN USA

**Keywords:** Metabolomics, Cognitive decline, Mobility decline, Aging, Dementia

## Abstract

Experiencing decline in both cognition and mobility is associated with a substantially higher dementia risk than cognitive decline only. Metabolites associated with both cognitive and mobility declines may be early predictors of dementia and reveal specific pathways to dementia. We analyzed data from 2450 participants initially free of dementia who had 613 metabolites measured in plasma in 1998–1999 (mean age = 75.2 ± 2.9 years old, 37.8% Black, 50% women) from the Health, Aging and Body Composition study. Dementia diagnosis was determined by race-specific decline in 3MS scores, medication use, and hospital records through 2014. Cognition and mobility were repeatedly measured using 3MS and a 20-m walking test up to 10 years, respectively. We examined metabolite associations with changes in 3MS (*n* = 2046) and gait speed (*n* = 2019) using multivariable linear regression adjusted for age, sex, race, and baseline performance and examined metabolite associations with dementia risk using Cox regression. During a mean follow-up of 9.3 years, 534 (21.8%) participants developed dementia. On average, 3MS declined 0.47/year and gait declined 0.04 m/sec/year. After covariate adjustment, 75 metabolites were associated with cognitive decline, and 111 metabolites were associated with gait decline (FDR-adjusted *p* < 0.05). Twenty-six metabolites were associated with both cognitive and gait declines. Eighteen of 26 metabolites were associated with dementia risk (*p* < 0.05), notably amino acids, glycerophospholipids (lysoPCs, PCs, PEs), and sphingolipids. Results remained similar after adjusting for cardiovascular disease or apolipoprotein E ɛ4 carrier status. During aging, metabolomic profiles of cognitive decline and mobility decline show distinct and shared signatures. Shared metabolomic profiles suggest that inflammation and deficits in mitochondria and the urea cycle in addition to the central nervous system may play key roles in both cognitive and mobility declines and predict dementia. Future studies are warranted to investigate longitudinal metabolite changes and metabolomic markers with dementia pathologies.

**Supplementary Information:**

The online version contains supplementary material available at 10.1007/s11357-024-01228-7.

## Introduction

Declines in mobility and cognition are common in aging, and both are critical to one’s quality of life and independence in late life. Biomarkers associated with cognitive decline and mobility decline may provide clues about the mechanisms underlying these aging phenotypes. Dual decline in mobility and cognition is associated with a substantially higher dementia risk than cognitive decline only [1–3]. Shared biomarkers may be important early predictors of dementia and may explain the biological underpinnings of the connection between dual decline and high dementia risk [4].

There is growing evidence that the study of blood-based omics data, such as metabolomics, can contribute to understanding mechanisms underlying aging phenotypes. Metabolomics quantifies small molecules that in aggregate suggest that specific biological processes and pathways contribute to the development of a specific phenotype. Specific metabolites and pathways have been related to cognition and gait speed, but data are mostly from cross-sectional studies. Evidence to date suggests that sphingolipids, sphingolipid metabolism pathway, biosynthesis of the unsaturated fatty acids and amino acids, and the kynurenine pathway may contribute to both cognition and mobility impairments in aging [5–12]. These markers and pathways are related to apoptosis and proliferation, inflammation, protein synthesis, neurotransmitters, and mitochondrial dysfunction. However, studies often examined metabolomic markers of cognition and mobility separately. Only a few recent studies examined metabolomic associations with mobility and cognitive decline simultaneously, but how these metabolites were related to dementia risk are not further explored [13, 14].

In this longitudinal study of community-dwelling older adults initially free of dementia, we aimed to identify metabolomic markers of cognitive decline and mobility decline over a decade of follow-up and test the hypothesis that a group of shared metabolomic markers exist that predict future dementia.

## Methods

### Study populations

Study participants were drawn from the Health, Aging, and Body Composition (Health ABC) study. The Health ABC study is a study focusing on functional decline that began in 1997–1998 with annual follow-up visits, enrolling 3075 participants aged 70–79 years at study entry from two study sites, Pittsburgh PA, and Memphis, TN. Inclusion criteria at study entry included no reported difficulty walking a quarter mile, climbing up ten steps, not reporting disability in basic activities of daily living, no use of a walking aid, no history of cancer treatment in the past 3 years, and no plan to move out of the study area within 3 years after recruitment. Plasma metabolomics were assayed by the Broad Institute (Cambridge, MA) using plasma samples collected at year 2 visit (1998–1999) from 2469 participants. We excluded 19 individuals with prevalent dementia from the sample for metabolomics, leaving 2450 participants in this analysis. Of these participants, we examined metabolomic profiles of cognitive decline among those who had repeated measures of 3MS (*n* = 2046) and metabolomic profiles of mobility decline in those with repeated measures of usual gait speed (*n* = 2019) since year 1 up to year 10 visits. The institutional review boards of the University of Pittsburgh, the University of Tennessee, the University of California-San Francisco Coordinating Center, and the National Institute on Aging approved the Health ABC study. All participants provided written informed consent at each visit.

### Plasma metabolomics

Plasma metabolomics were assayed using liquid chromatography-mass spectrometry (LC–MS). Blood samples at the year 2 visit in 1998–1999 were drawn after overnight fasting and were stored at − 80 ℃ until metabolite profiling in 2019 − 2021. Details of the metabolite profiling were previously published [15, 16]. Four complimentary LC–MS methods were used, measuring (1) lipids (C8-positive); (2) a variety of amino acids, acylcarnitines, dipeptides, and other cationic polar metabolites (hydrophilic interaction liquid chromatography [HILIC]-positive); (3) fatty acids, eicosanoids, and bile acids (C18-negative); and (4) sugars, organic acids, nucleotides, and anionic polar metabolites (HILIC-negative). Because four LC–MS methods were used to quantify metabolites, metabolites could be measured in more than one platform. In this analysis, we analyzed all 613 available metabolites including 520 unique metabolites. Missing values of the metabolites considered below the limit of detection were imputed using half of the minimum. Metabolite values were then log-transformed and computed as standard *Z* scores for analysis.

### Cognitive function and mobility

Global cognitive status was measured using the Modified Mini-Mental State Exam (3MS) at years 1, 3, 5, 8, and 10. Mobility was measured using a 20-m walk test at years 1, 2, 3, 4, 5, 6, 8, and 10.

### Dementia diagnosis

Based on a previously developed algorithm in the Health ABC study, dementia was determined by hospital records, the use of prescribed medications for dementia (galantamine, rivastigmine, memantine, donepezil, or tacrine), or race-specific decline in 3MS greater than 1.5 SDs from the baseline mean [17].

### Statistical analysis

To determine metabolomic markers of cognitive decline and gait decline, we examined the associations between each metabolite and slopes of 3MS and slopes of gait speed, separately, using multivariable linear regression, adjusted for age, sex, race, and baseline 3MS and baseline gait speed, respectively. Slopes of 3MS and gait speed were first computed using linear mixed-effects model using all available data since year 1 up to year 10 visits. To understand whether metabolomic markers of baseline performance and change over time share similarities, we also examined the associations between each metabolite and baseline 3MS and baseline gait speed using multivariable linear regression, adjusted for age, sex, and race. The significance for metabolite associations with gait and cognition was set at FDR-adjusted *p* < 0.05.

For metabolites that were significantly associated with both gait decline and cognitive decline, we further examined their associations with dementia risk using Cox regression, adjusted for age, sex, and race, and additionally adjusted for the prevalence of cardiovascular disease at metabolomics assessment and apolipoprotein E (APOE) ε4 carrier status in those with available data (*n* = 2328). Significance for specific metabolite associations with dementia risk was set at *p* < 0.05.

To understand whether metabolomic markers of cognition were correlated with metabolomic markers of gait speed, we created metabolite scores using the least absolute shrinkage and selection operator (LASSO) models and tested the associations between LASSO metabolite scores. We also performed pathway analysis when there was sufficient information on the human metabolomic database (HDMB) identification numbers matching the current database via MetaboAnalyst.ca. Significance for associations between LASSO metabolite scores and pathway analysis was reported at *p* < 0.05. All analyses were performed using R studio 4.3.1 (Boston, MA).

## Results

Participants’ characteristics at the time of metabolomics assessment are presented in Table 1. The participants, including 50% women, were in their 70 s with a relatively narrow age range between 69.4 and 82.0 years; 37.8% were Black, 76% had greater than high school education, and 28.9% were APOE ε4 carriers. During a mean follow-up of 9.3 years, 534 (21.8%) participants developed dementia. The incidence rate was 23.4 per 1000 person-years.

**Table 1:** Participants’ characteristics at year 2 metabolomics assessment (*n* = 2450)

	Mean ± SD or *N* (%)	Range
Demographics		
Age, years	75.2 ± 2.9	69.4 to 82.0
Men	1225 (50%)	-
Black	927 (37.8%)	-
Greater than high school education	1864 (76%)	-
Body mass index, kg/m^2^	27.2 ± 4.8	14.1 to 50.9
Apolipoprotein E ε4 carrier status	673 (28.9%)	-
Functional measures		
Baseline 3MS, unitless	91 ± 7 (*n* = 2046)	29 to 100
Slope of 3MS decline, per year	− 0.47 ± 0.71 (*n* = 2046)	− 6.26 to 1.07
Baseline gait speed, m/sec	1.35 ± 0.25 (*n* = 2019)	0.38 to 2.47
Slope of gait decline, m/sec/year	− 0.04 ± 0.01 (*n* = 2019)	− 0.088 to 0.010
Incident dementia	534 (21.8%)	-
Disease conditions		
Cardiovascular disease	707 (28.9%)	-
Hypertension	1318 (53.8%)	-
Diabetes	459 (18.7%)	-
Cancer (except non-melanoma skin cancer)	479 (19.6%)	-
Depression	235 (9.6%)	-
Peripheral artery disease*	123 (5.0%)	-
Hip or knee osteoarthritis*	94 (3.8%)	-
Gall stones*	326 (13.3%)	-
Abdominal wall hernia*	415 (17.0%)	-
Gastrointestinal bleed*	246 (10.1%)	-
Stomach/duodenal ulcer*	386 (15.9%)	-
Pulmonary disease*	279 (11.4%)	-

Note: *3MS*, Modified Mini-Mental State Exam. *Prevalence at study entry year 1 visit. The prevalence of these conditions is not available at year 2 visit

### Metabolites with cognition

Over the 10-year follow-up, the average decline of 3MS was 0.47 points per year (Table 1). Of the 613 metabolites examined, 70 metabolites were associated with baseline 3MS, and 75 metabolites were associated with change in 3MS up to 10 years (FDR-adjusted *p* < 0.05) (Table 2, Supplementary Table 1). Fifteen metabolites were associated with both baseline 3MS and decline in 3MS and showed consistent directions. Of these, nine metabolites were positively associated with baseline 3MS and change in 3MS, and six metabolites were negatively associated with baseline 3MS and change in 3MS. These 15 metabolites included amino acids (alpha-N-Phenylacetylglutamine via both C18n and HILICp platforms, homocitrulline, N-Methyl-proline, tryptophan), carbohydrates (P-Cresol glucuronide, glyceric acid), phospholipids (LPC (22:5), SM (d18:1/14:0 via C8p and HILICp platforms), pyrimidines (uracil, uridine), fatty acids (myristoleic acid), prenol lipids (maslinic acid), and steroids (CE(14:0) (FDR-adjusted *p* < 0.05). Some metabolites were implicated in pathways of pyrimidine metabolism (uracil, uridine), glycerolipid metabolism (glyceric acid), and pantothenate and CoA biosynthesis (uracil) (*p* < 0.05). The LASSO metabolite score of baseline 3MS was associated with the LASSO metabolite score of changes in 3MS (*β* = 0.308, *p* < 0.001) (*n* = 2046) (Supplementary Fig. 1).

**Table 2:** Associations between 26 shared metabolites of cognitive decline and gait decline and dementia risk

Metabolites	Cognitive decline (*n* = 2046)		Gait decline (*n* = 2019)		Incidence of dementia (*n* = 2450)		HMDB analyte class	Sub class	HMDB ID
	*β*	FDR-adj. *p* value	*β*	FDR-adj. *p* value	HR	*p* value			
Tryptophan	0.050	0.026	0.001	0.005	0.892	0.012	Carboxylic acids and derivatives	Amino acids, peptides, and anal	0000929
alpha-N-phenylacetylglutamine (HILIC)	− 0.055	0.007	− 0.001	0.034	1.138	0.005	Carboxylic acids and derivatives	Amino acids, peptides, and analogues	0006344
Uracil	0.077	0.000	0.001	0.045	0.854	0.000	Diazines	Pyrimidines and pyrimidine derivatives	0000300
Adrenic acid	− 0.061	0.002	− 0.001	0.032	1.137	0.005	Fatty acyls	Fatty acids and conjugates	0002226
Eicosenoic acid	− 0.065	0.001	− 0.001	0.017	1.102	0.034	Fatty acyls	Fatty acids and conjugates	0002231
CAR (4:0)	− 0.044	0.034	− 0.001	0.001	1.082	0.076			
TG (54:9)	0.049	0.020	0.001	0.012	0.945	0.202			
TG (56:10)	0.055	0.007	0.001	0.015	0.918	0.054			
LPC (14:0)	0.051	0.023	0.001	0.006	0.925	0.098			
LPC (20:5) (C8p)	0.061	0.002	0.001	0.000	0.893	0.012	Glycerophospholipids	Glycerophosphocholines	0010397
LPC (22:6)	0.050	0.013	0.001	0.003	0.945	0.205			
PC (38:6)	0.066	0.001	0.001	0.005	0.905	0.030	Glycerophospholipids	Glycerophosphocholines	0007991
PC (40:6)	0.063	0.002	0.001	0.011	0.900	0.024	Glycerophospholipids	Glycerophosphocholines	0008057
PC (40:9)	0.063	0.001	0.001	0.008	0.899	0.019	Glycerophospholipids	Glycerophosphocholines	0008731
PC (P-38:5)/PC (O-38:6) (C8p)	0.080	0.000	0.001	0.045	0.831	0.000	Glycerophospholipids	Glycerophosphocholines	0011319
PE (32:0)	0.050	0.025	0.001	0.016	0.940	0.170			
PE (34:0)	0.050	0.021	0.001	0.001	0.927	0.099			
PE (P-36:0)/PE (O-36:1)	0.044	0.030	0.001	0.000	0.869	0.001	Glycerophospholipids	Glycerophosphoethanolamines	0009016
PE (P-38:6)/PE (O-38:7) (C8p)	0.066	0.001	0.001	0.017	0.854	0.001	Glycerophospholipids	Glycerophosphoethanolamines	0011420
PE (P-40:6)/PE (O-40:7) (C8p)	0.059	0.007	0.001	0.020	0.850	0.001	Glycerophospholipids	Glycerophosphoethanolamines	0011394
PS (34:0)	0.070	0.000	0.001	0.002	0.866	0.001	Glycerophospholipids	Glycerophosphoserines	0012356
Maslinic acid	0.070	0.000	0.001	0.045	0.882	0.007	Prenol lipids	Triterpenoids	0002392
N2,N2-dimethylguanosine	− 0.048	0.030	− 0.001	0.001	1.058	0.236			
SM (d18:1/14:0) (C8p)	0.069	0.001	0.001	0.000	0.834	0.000	Sphingolipids	Phosphosphingolipids	0012097
SM (d18:1/14:0) (HILICp)	0.045	0.046	0.001	0.015	0.872	0.003	Sphingolipids	Phosphosphingolipids	0012097
CE (20:5)	0.053	0.007	0.001	0.024	0.894	0.014	Steroids and steroid derivatives	Steroid esters	0006731

Note: The bold number indicates *p* value < 0.05 for metabolite associations with incident dementia

### Metabolites with gait speed

Over the 10-year follow-up, the average decline in gait speed was 0.04 m/sec per year. There were 197 metabolites associated with baseline gait speed, and 111 metabolites were associated with change in gait speed up to 10 years (FDR-adjusted *p* < 0.05) (Table 2, Supplementary Table 1). There were 57 metabolites associated with both baseline gait speed and gait decline and showed consistent directions for cross-sectional and longitudinal associations. Of these, 30 metabolites were positively associated with baseline gait speed and change in gait speed, and 27 were negatively associated with baseline gait speed and change in gait speed. These 57 metabolites mainly included glycerophospholipids, amino acids, carbohydrates, fatty acids, and triacylglycerols. Of these 57, metabolites from glycerophospholipids (lysoPCs, PCs), triacylglycerols (TGs), some amino acids (homoarginine, tryptophan), and pyrimidines (uracil) were all positively associated with gait speed or change in gait speed (i.e., upregulated), and metabolites from carbohydrates (such as glucuronic acid, threitol) and fatty acids (such as CAR, adrenic acid, eicosenoic acid) and some amino acids (such as alpha-N-phenylacetylglutamine) were all negatively associated with gait speed or change in gait speed (i.e., downregulated). Some of these metabolites were implicated in pantothenate and CoA biosynthesis, citrate cycle (TCA cycle), beta-alanine metabolism, and pyrimidine metabolism (*p* < 0.05). The LASSO metabolite score of baseline gait speed was associated with the LASSO metabolite score of change in gait speed (*β* = 0.470, *p* < 0.001) (*n* = 2019) (Supplementary Fig. 2).

### Shared metabolites of cognitive decline and gait decline with dementia risk

**Fig. 1:**
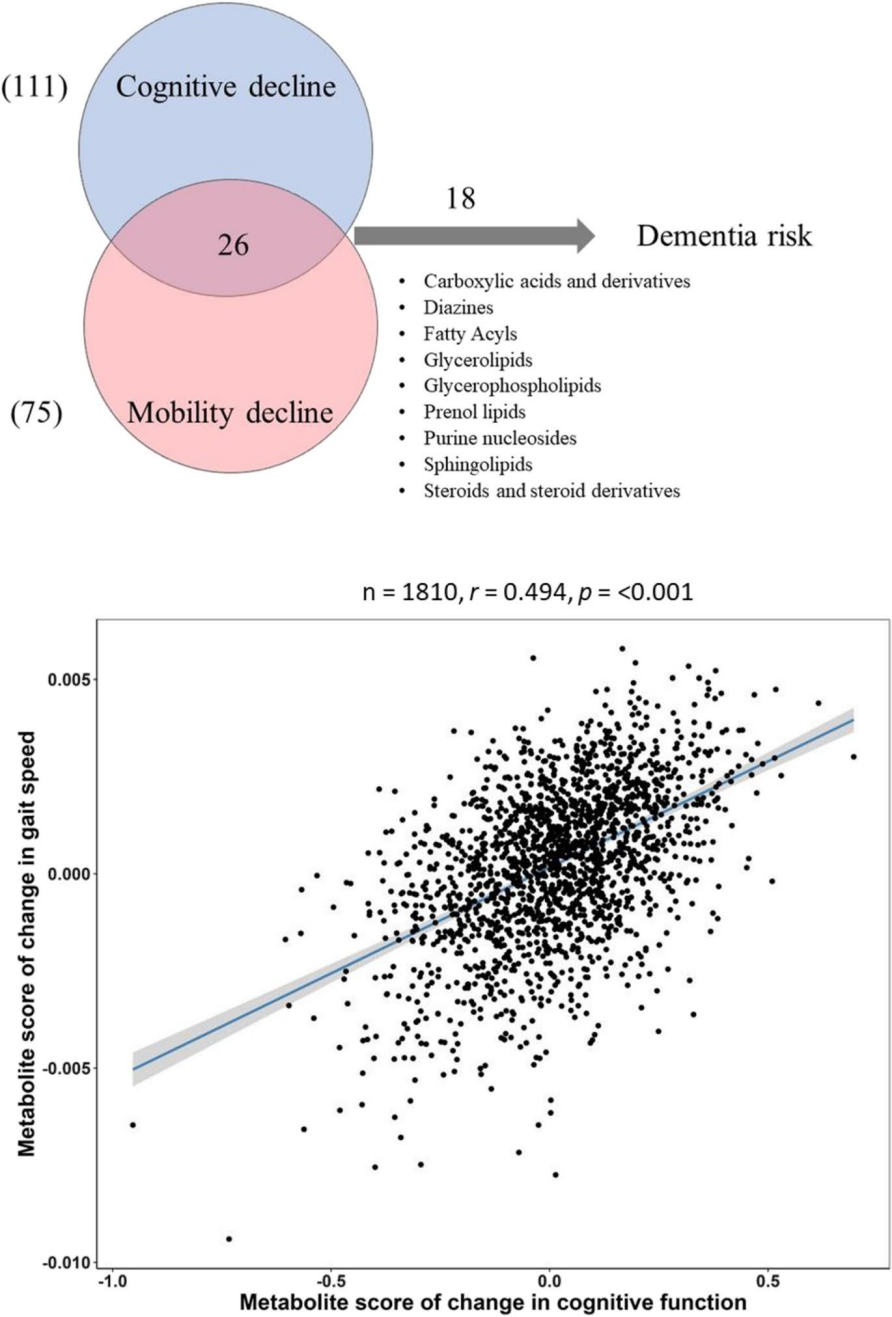
Metabolites associated with gait decline, cognitive decline, and dementia risk. **a** Number of metabolites associated with cognitive decline, gait decline, and incident dementia. **b** Scatter plot of the association between the LASSO metabolite score of cognitive decline (*x*-axis) and LASSO metabolic score of gait decline (*y*-axis)

There were 26 metabolites associated with both change in 3MS and change in gait speed in consistent directions (FDR-adjusted *p* < 0.05) (Table 2) (Fig. 1). Most metabolites were positively associated with change in 3MS and change in gait speed, including glycerophospholipids (lysoPCs, PCs), sphingolipids (SM (d18:1/14:0), amino acids (tryptophan), and pyrimidines (uracil). Five metabolites were negatively associated with change in 3MS and change in gait speed, including fatty acids (CAR (4:0), adrenic acid, eicosenoic acid), amino acids (alpha-N-phenylacetylglutamine), and N^2^,N^2^-dimethylguanosine (Table 2). The LASSO metabolite score of change in 3MS was associated with the LASSO metabolite score of change in gait speed (*β* = 0.494, *p* < 0.001) (*n* = 1810) (Fig. 1).

**Fig. 2:**
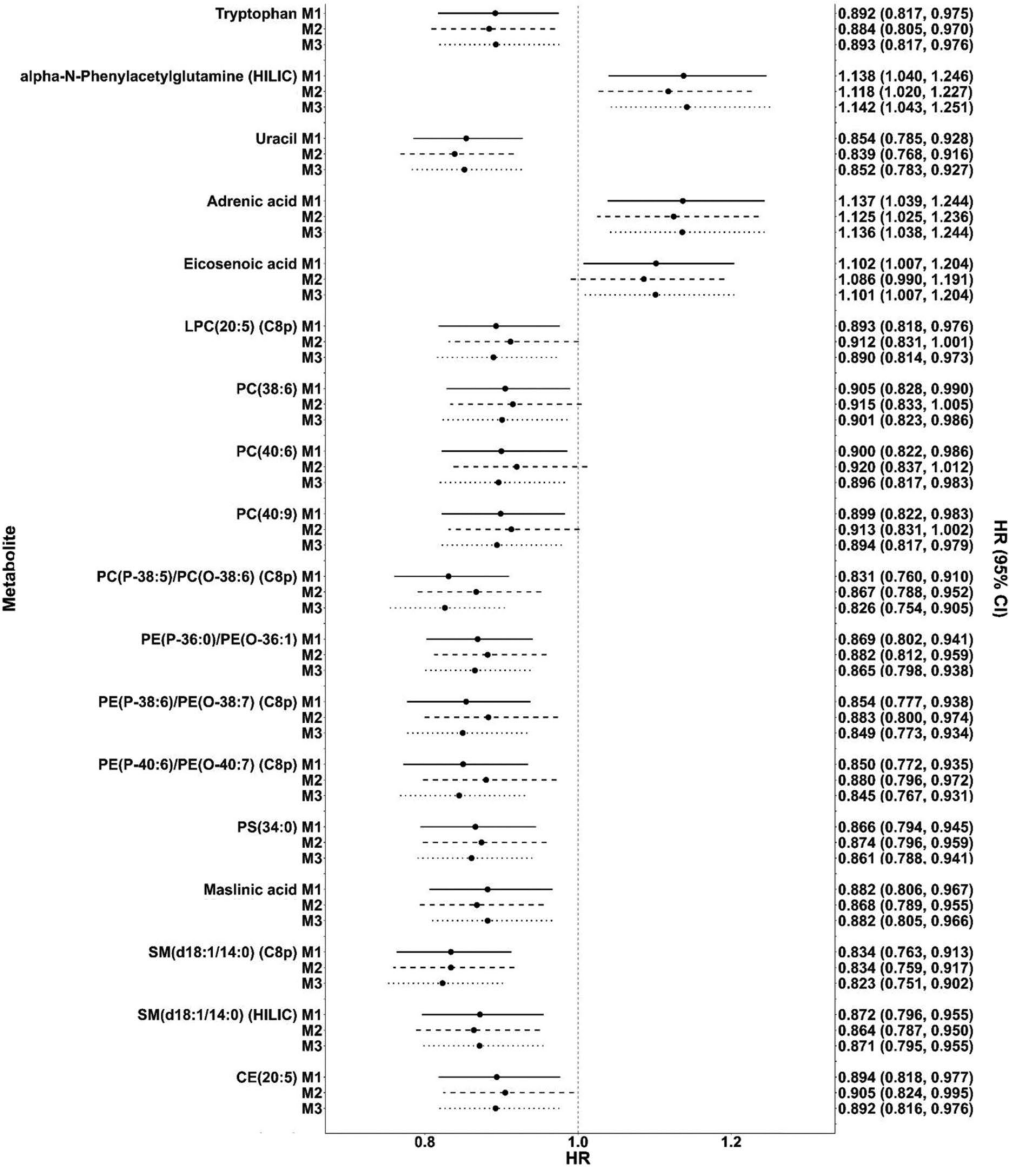
Dot plots of associations between 18 shared metabolomic markers of cognitive decline and gait decline and incident dementia with additional adjustment for apolipoprotein E ɛ4 (APOE ɛ4) status and cardiovascular disease. Legend: M1-covariate adjustment results same as Table 2. M2-additional adjustment for APOE ɛ4 carrier status. M3-additional adjustment for cardiovascular disease

Of these 26 metabolites, 18 were significantly associated with dementia risk (*p* < 0.05) (Table 2). Most of these metabolites were upregulated; higher concentrations were associated with a reduced risk of dementia, including lysoPC (20:5), sphingomyelins, tryptophan, uracil, CE (20:5), PCs, PEs, and PS. Other metabolites, including adrenic acid, eicosenoic acid, and alpha-N-phenylacetylglutamine, were downregulated; higher concentrations were associated with an increased risk of dementia. Results remained similar after further adjustment for APOE ɛ4 status or prevalence of cardiovascular disease at metabolomics assessment, while adjustment for APOE ɛ4 status attenuated some associations more than adjustment for cardiovascular disease (Fig. 2).

## Discussion

In this biracial cohort of community-dwelling older adults, we identified metabolomic markers of longitudinal cognitive decline and gait speed decline over a decade. Metabolomic profiles of cognitive decline and mobility decline show both unique and shared biomarkers. Some of the shared markers predicted dementia risk which provide additional insights into mechanisms underlying dual cognitive and mobility decline in the development of dementia.

Among all the 613 metabolites examined, we identified 75 metabolites associated with cognitive decline after adjustment for baseline cognitive function. The metabolomic profiles are mainly derived from amino acids, fatty acids, glycerophosphocholines, and phosphosphingolipids. We confirmed some of these metabolites which were previously reported in either cross-sectional or longitudinal studies of cognitive function, such as alpha-N-phenylacetylglutamine, tryptophan, and PC (38:6) [5]. Alpha-N-phenylacetylglutamine is the main amino acid from the phenylacetate degradation in the urea cycle and high levels of which are implicated in urea cycle disorders, digestive disorders, and deficits in the nervous system. Tryptophan is an essential alpha-amino acid that cannot be synthesized de novo. It is one of the precursors to the neurotransmitter serotonin and of the nicotinamide adenine dinucleotide (NAD +) and nicotinamide adenine dinucleotide phosphate (NADP +), which are essential co-factors in bioenergetic and biosynthesis pathways. PC (38:6) is a glycerophospholipid that includes palmitic and docosahexaenoic fatty acids, which are particularly abundant in fish oils. PC (38:6) has been identified in vascular disorders, digestive system disorders, and other conditions. We identified additional metabolites related to cognitive decline which have not been reported before. For instance, other amino acids (such as homocitrulline), fatty acids (such as adrenic acid, arachidic acid, eicosenoic acid, oleic acid, and stearic acid), other glycerophosphocholines (such as lysoPCs, PCs, LPEs, and PEs), phosphosphingolipids (SM (d18:1/14:0) and S1P), pyrimidines (such as uracil and uridine), maslinic acid, serotonin, carboxyibuprofen, and sphingosine 1-phosphate were also associated with cognitive decline. Uridine and uracil are essential components of DNA and RNA, important to maintain genetic integrity and active transcription of enzyme and signaling molecules [18]. Adrenic acid is a polyunsaturated fatty acid and is involved in alpha-linolenic acid and linoleic acid metabolism. It is abundant in human brains during early development, and some evidence suggests that oxidation of adrenic acid may contribute to cerebral white matter damage [19]. Oleic acid is classified as a monounsaturated omega-9 fatty acid and is the most abundant fatty acid in human adipose tissue. Maslinic acid is a pentacyclic triterpene and shown to have anti-inflammatory, antioxidant, and neuroprotective effects [20]. It is abundant in olives [21, 22] and can also be found in other vegetables and fruits, such as loquat leaves [23], red dates [24, 25], and plantains [26]. SM (d18:1/14:0) is a sphingosine backbone with a myristic acid chain. It is especially found in myelin sheaths surrounded by nerve cell axons. Serotonin is a major neurotransmitter involved in various physiological processes, including digestion, mood, and sleep. Ibuprofen is commonly used to treat pain and reduce inflammation. Higher levels of carboxyibuprofen being associated with greater cognitive decline may indicate the presence of inflammation. Sphingosine 1-phosphate, which delays cellular senescence, is reportedly associated with gait performance [8]. We now found it was also associated with cognitive decline. Taken together, metabolomic profiles of cognitive decline point to the dysregulation of the urea cycle, protein synthesis, and central nervous system (CNS). Among the 75 metabolites associated with cognitive decline, 15 were also associated with baseline cognition and showed consistent directions of upregulation and downregulation.

Nearly half of the metabolomic profiles of gait decline are glycerophosphocholines and glycerophosphoethanolamines, including lysoPCs, PCs, and PEs. Specific lysoPCs, such lysoPC (16:0), (16:1), (18:0), (18:1), and (18:2), are consistent with previous findings [5, 14, 27]. These metabolites are important precursors of cardiolipin which is the main structure of the mitochondrial inner member and have been associated with skeletal muscle mitochondrial function [28]. We also confirmed other metabolites, such as N^2^,N^2^-dimethylguanosine, N-formylmethionine, and hexose [5, 29]. N^2^,N^2^-dimethylguanosine is a methylated purine metabolite derived from tRNA degradation and has been involved in renal dysfunction [30]. N-formylmethionine plays an effective role in initiating protein synthesis. The process of methionine entering the ribosome occurs in the mitochondria of eukaryotic cells, Escherichia coli, and other bacteria [31]. Recent data from critically ill patients have shown that circulating N-formylmethionine is related to metabolic shift and high mortality which may involve impaired mitochondrial oxidation, elevated branch chain amino acid metabolism, and triggering the pentose phosphate pathway [32]. Multiple sugars are classified under the label “Hexose” including glucose, galactose, and fructose, which may suggest that high levels of sugar intake or circulating glucose contribute to gait decline. The remaining profiles are mainly from amino acids, carbohydrates, and sphingomyelins. Some of these are shared with metabolomic profiles of cognitive decline, such as tryptophan, alpha-N-phenylacetylglutamine, PC (38:6), SM (d18:1/14:0), and maslinic acid. We found additional metabolites associated with gait decline which were not reported nor investigated before. Some of the metabolites may suggest the important roles of metabolic disorders and vascular problems in gait decline. For instance, metformin and warfarin are used to treat diabetes and prevent blood clots, respectively. The negative associations of these metabolites with gait decline may indicate the presence of diabetes and heart disease. Threitol, PE (P-36:0)/PE (O-36:1), and SM (d18:1/14:0), are reportedly involved in diabetes. Sulfamethoxazole is an antibacterial and antibiotic agent and showed a positive association with gait decline. The role of sulfamethoxazole in physical function or frailty is unclear in humans, while some animal data shows that antibiotics increases locomotion [33].

The metabolomic profiles of cognitive decline and gait decline share 24 metabolites from amino acids, carbohydrates, carboxylic acids, glycerophosphocholines, and phosphosphingolipids. These shared profiles are in line with recently published data on metabolomic changes of dual decline in cognition and mobility which suggests deficits in mitochondrial function, compromised immunity, and increased burden in cardiovascular system and kidney [13, 14]. Specific lysoPCs and maslinic acid may highlight the importance of mitochondria function and inflammation. Alpha-N-phenylacetylglutamine may suggest the important role of the urea cycle. N^2^,N^2^-dimethylguanosine and uracil may indicate the importance of RNA synthesis and degradation. Other metabolites, such as SM (d18:1/14:0), adrenic acid, and tryptophan, may suggest the key role of the CNS in both cognitive decline and gait decline. The LASSO metabolite scores of cognitive decline and gait decline show a moderate correlation, suggesting substantial overlapping metabolomic markers predicting both cognitive decline and gait decline. We further identified that the majority of shared metabolomic markers predicted dementia risk, and further adjustment for the APOE e4 genotype did not substantially alter these associations. These findings provided additional insights into the connection between dual decline and high risk of dementia.

This study has novel aspects, and we extended prior literature from several aspects. First, metabolomic markers covered more than 600 metabolites, some of which were measured on more than one platform. This provides a more comprehensive look than examining only a few metabolites, and finding the same metabolites in more than one platform adds robustness to the findings. Second, multiple repeated assessments of cognitive function and gait speed over a decade allow us to more accurately model functional decline than only one-time assessment, two, or a few repeated measures. Third, the study population is diverse and includes nearly 40% Black participants. Last but not the least, we extended prior research by identifying shared metabolomic markers with dementia risk. One potential limitation is the dementia diagnosis based on cognitive decline, hospital records, and medication use. The ascertainment may be less rigorous compared to using cognitive adjudication conferences conducted in other studies.

In conclusion, among older adults initially free of dementia, metabolomic profiles of cognitive decline and mobility decline show distinct and shared signatures. Metabolites key to the urea cycle, CNS especially neurotransmitters and myelin health, protein synthesis, and inflammation are associated with cognitive decline; and metabolites important for mitochondrial function, urea cycle, carbohydrate metabolism, diabetes, and metabolic disorders are associated with gait decline. Shared metabolomic profiles which suggest mitochondrial function, inflammation, and urea cycle in addition to CNS may play key roles in both cognitive and mobility declines and predict dementia risk. These findings warrant validation in other aging populations. Future studies are warranted to understand these shared metabolomic markers with pathologies underlying dementia and subtypes and to investigate longitudinal relationships of metabolomic changes with functional decline.

### Supplementary Information

Below is the link to the electronic supplementary material.

Supplementary file1 (DOCX 641 KB)
