# Supplementary material for: Shared plasma metabolomic profiles of cognitive and mobility decline predict future dementia
Source: GeroScience. 2024 Jun 3;46(5):4883–94. doi: 10.1007/s11357-024-01228-7 (PMC11336156; doi:10.1007/s11357-024-01228-7)
Supplement: Supplementary file 1 — Supplementary file1 (DOCX 641 KB) [file 11357_2024_1228_MOESM1_ESM.docx]

**Supplementary Table 1. Metabolites associated with cognitive decline and gait decline**

| **Metabolites associated with cognitive decline** | **Metabolites associated with gait decline** | **Human Metabolome Database Sub-Class** |
| --- | --- | --- |
| alpha-N-phenylacetylglutamine (C18n), alpha-N-Phenylacetylglutamine (HILICp), 2-Aminobutyric acid, aminoadipic acid, homocitrulline, N-Methyl-proline, proline betaine, tryptophan | N-Formylmethionine, Valsartan, 4-Acetamidobutanoic acid, Betaine, Glutamic acid, Guanidoacetic acid, Histidine, Homoarginine, Lysine, Tryptophan, alpha-N-Phenylacetylglutamine | Amino acids, peptides, and analogues |
|  | Sulfamethoxazole | Benzenesulfonamides |
|  | 4-Aminohippuric acid | Benzoic acids and derivatives |
|  | Bilirubin | Bilirubins |
| P-Cresol glucuronide, glyceric acid, malonic acid | Glucuronic acid, Hexose, Sucrose or Lactose or Trehalose, Threitol, Fumaric acid or Maleic acid, cis-Aconitic acid N-Acetylputrescine,  N^1^,N^12^-Diacetylspermine | Carbohydrates, carbohydrate conjugates, and dicarboxylic acids and derivatives, Tricarboxylic acids and derivatives, Carboximidic acids |
|  | Cholesterol | Cholestane steroids |
|  | Phytanic acid | Diterpenoids |
|  | 3-Dehydroxycarnitine | Furanocoumarins |
|  | Metformin | Guanidines |
|  | Warfarin (C18n), Warfarin (HILICp) | Hydroxycoumarins |
|  | MG(18:0) | Monoacylglycerols |
|  | DMGV | Short-chain keto acids and derivatives |
| CAR(18:1), CAR(4:0), CAR(DC5:0) | CAR(4:0), CAR(6:0) | Fatty acid esters |
| 10-HDoHE, sebacic acid (C18n), sebacic acid (HILICn), myristoleic acid, 11Z,14Z-Eicosadienoic acid, 12,13-DiHOME, adrenic acid, arachidic acid, eicosenoic acid, oleic acid, stearic acid | adrenic acid, eicosenoic acid | Fatty acids and conjugates |
| LPC(14:0), LPC(20:5), LPC(22:5), LPC(22:6), LPC(18:1) (HILICp),  PC(36:4-OH), PC(38:6), PC(40:6), PC(40:9), PC(P-38:5)/PC(O-38:6), PC(P-38:6)/PC(O-38:7),  LPE(18:1), LPE(20:4)  PE(32:0),PE(34:0),PE(P-36:0)/PE(O-36:1),PE(P-38:6)/PE(O-38:7),PE(P-40:6)/PE(O-40:7) | LPC 15:0, LPC 17:0, LPC 17:1, LPC(P-16:0)/LPC(O-16:1) (HILICp), LPC(P-18:0)/LPC(O-18:1)_A LPC(18:0) (C8p), LPC(18:0) (HILICp), LPC(18:1) C8p, LPC(14:0), LPC(16:0), LPC(18:2), LPC(20:0), LPC(20:1), LPC(20:4), LPC(20:5), LPC(22:6), LPC(24:0), LPC(P-16:0)/LPC(O-16:1) (C8p), LPC(P-18:0)/LPC(O-18:1)  PC(34:0),PC(34:4), PC(36:0), PC(36:4)_A, PC(36:4)_B, PC(38:4), PC(38:6), PC(40:6)  PC(40:9), PC(P-34:4)/PC(O-34:5), PC(P-36:4)/PC(O-36:5)_B, PC(P-38:5)/PC(O-38:6)  PC(P-34:1)/PC(O-34:2)  PC(P-34:2)/PC(O-34:3)  LPE(18:0),LPE(16:0),LPE(18:0)_A,LPE(18:0)_B,PE(32:0),  PE(34:0) (C8p), PE(36:0), PE(38:2), PE(34:0) (HILICp),LPE(20:0),LPE(22:0),PE(P-36:0)/PE(O-36:1),PE(P-38:2)/PE(O-38:3),PE(P-38:6)/PE(O-38:7),PE(P-40:6)/PE(O-40:7) | Glycerophosphocholines and  Glycerophosphoethanolamines |
| PS(34:0) | PS(34:0) | Glycerophosphoserines |
| N-Acetylcarnosine |  | Hybrid peptides |
| Cortisol |  | Hydroxysteroids |
| Indolelactic acid | 3-Indolepropionic acid | Indolyl carboxylic acids and derivatives |
| Homovanillic acid |  | Methoxyphenols |
| Carboxyibuprofen |  | NA |
| Uridine |  | NA |
| N^2^,N^2^-Dimethylguanosine | N^2^,N^2^-Dimethylguanosine | NA |
|  | Hydrocinnamic acid | NA |
|  | Glucosan or 3-Hydroxymethylglutaric acid | NA |
|  | Pseudouridine (HILICn), Pseudouridine (HILICp) | NA |
|  | 1-Methyladenosine | NA |
|  | 1-Methylguanosine | NA |
|  | Inosine | NA |
|  | N4-Acetylcytidine | NA |
| Taurine |  | Organosulfonic acids and derivatives |
| Sphingosine 1-phosphate, SM(d18:1/14:0) (C8p), SM(d18:1/14:0) (HILICp) | SM(d18:1/14:0) (C8p), SM(d18:1/14:0) (HILICp)  SM(d18:1/20:0), SM(d18:1/22:1) | Phosphosphingolipids |
| ADP, GDP, AMP, ATP |  | Purine ribonucleotides |
| Xanthine | 1-Methylxanthine (HILICn), 1-Methylguanine (HILICp) | Purines and purine derivatives |
| Niacinamide | Quinolinic acid | Pyridinecarboxylic acids and derivatives |
| Uracil | Uracil | Pyrimidines and pyrimidine derivatives |
| Choline phosphate |  | Quaternary ammonium salts |
| CE(14:0), CE(20:5) | CE(18:0), CE(20:5) | Steroid esters |
|  | Pregnenolone sulfate | Sulfated steroids |
| TG(54:9),TG(56:10),TG(56:7),TG(56:8),TG(58:10),TG(58:11),TG(58:9),TG(60:12) | TG(50:6), TG(52:7), TG(53:3), TG(54:9), TG(56:10) | Triacyclglycerols |
| Maslinic acid | Maslinic acid | Triterpenoids |
| Serotonin |  | Tryptamines and derivatives |
|  | Ureidopropionic acid | Ureas |


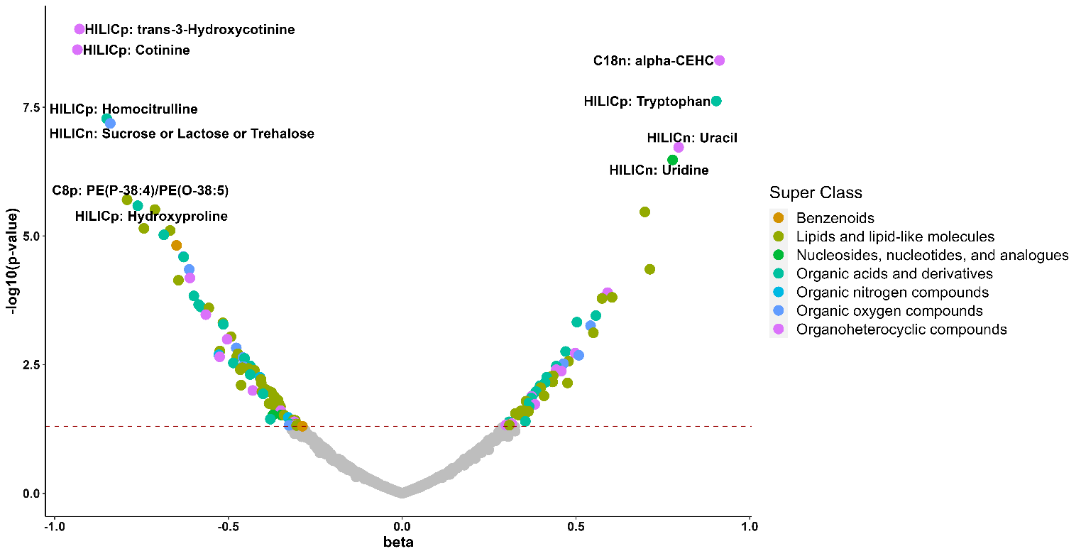

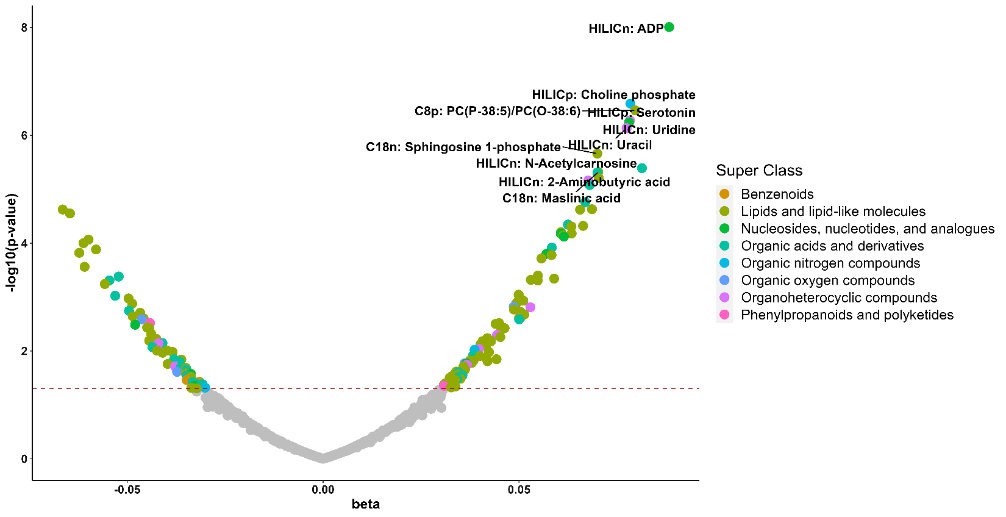


1. **Metabolites associated with baseline 3MS (b) Metabolites associated with changes in 3MS up to year 10 visit**
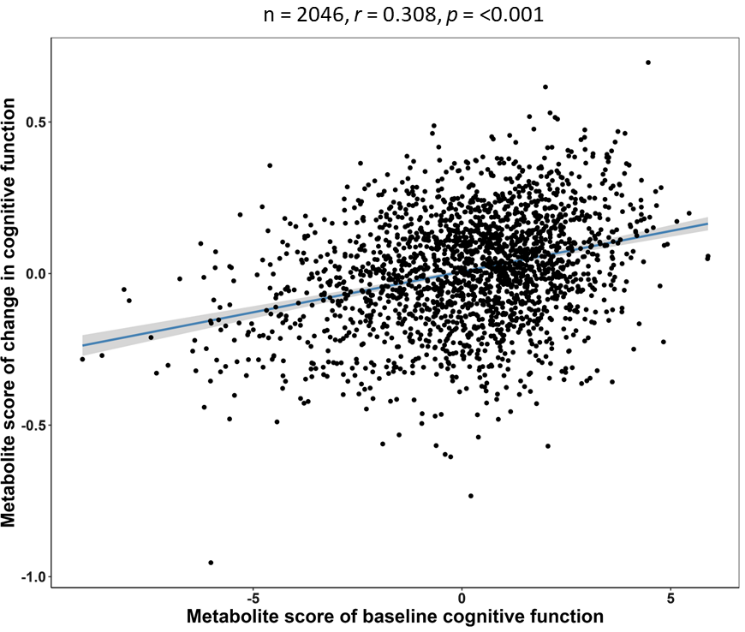


**(c)**

**Supplementary Figure 1. Metabolites that are associated with baseline cognitive function and change in cognitive function over time.**

(a) Volcano plot of metabolites associated with baseline 3MS. (b) Volcano plot of metabolites associated with change in 3MS since year 1 up to year 10 visits. Top 10 significant metabolites were labeled with metabolite names (c) Scatter plot of the association between the LASSO metabolite score of baseline 3MS (x-axis) and LASSO metabolite score of changes in 3MS (y-axis)


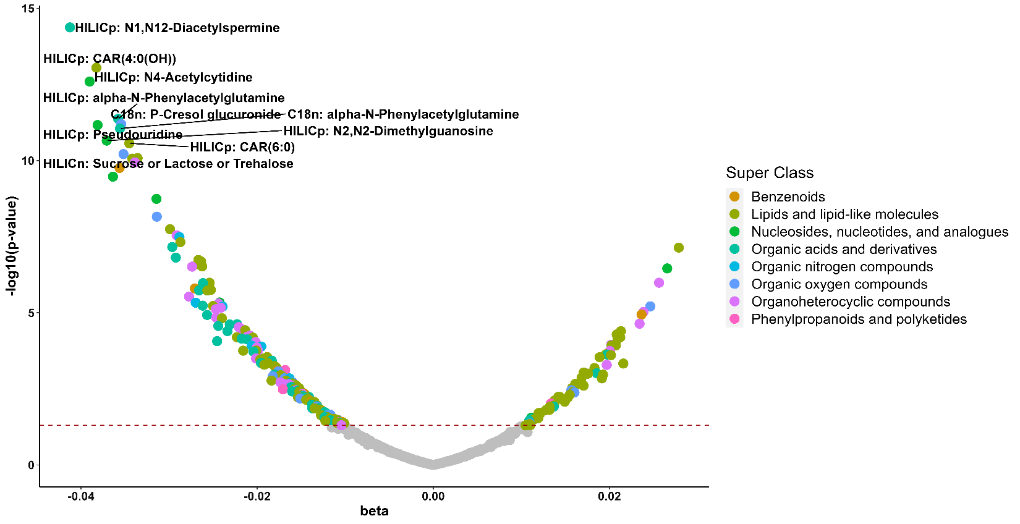

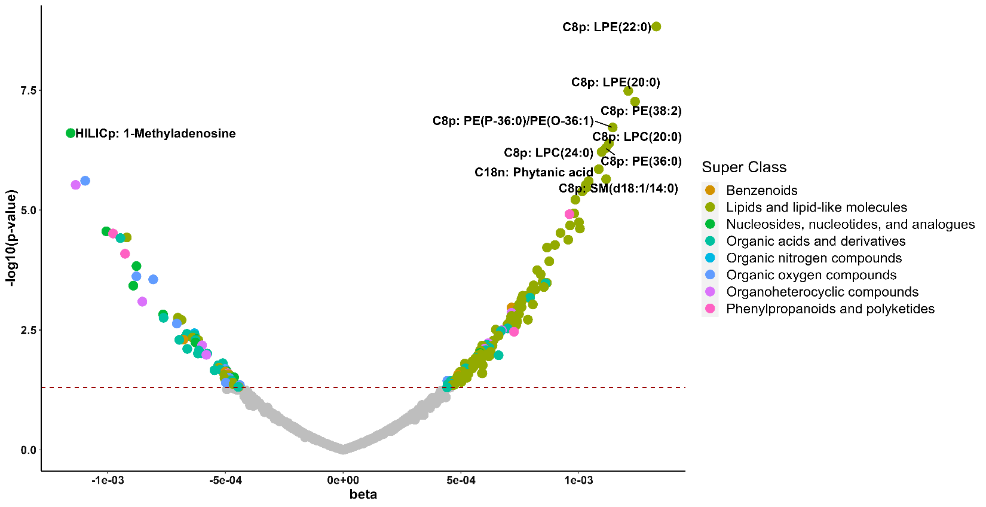


1. **Metabolites associated with baseline gait speed b) Metabolites associated with changes in gait speed up to year 10 visit**


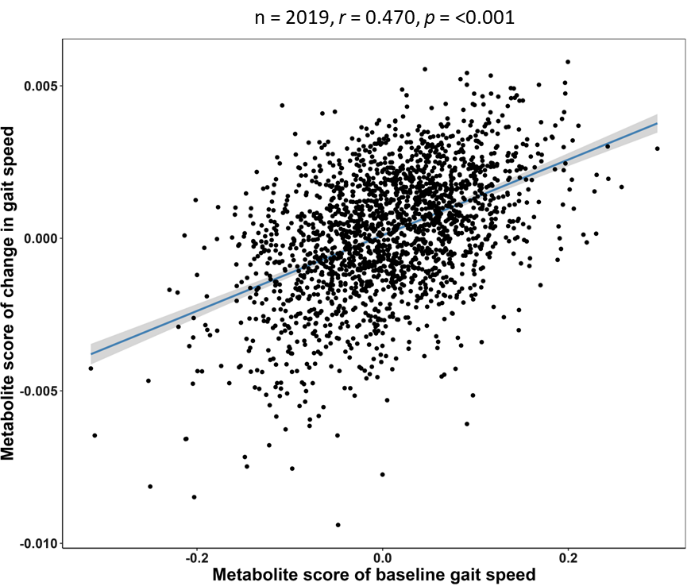


**(c)**

**Supplementary Figure 2. Metabolites associated with baseline gait speed and changes in gait speed.**

(a) Volcano plot of metabolites associated with baseline gait speed. (b) Volcano plot of metabolites associated with changes in gait speed since year 1 up to year 10 visits. Top 10 significant metabolites were labeled with metabolite names. (c) Scatter plot of the LASSO metabolite scores of baseline gait and LASSO metabolite score of changes in gait speed
